# Baroreflex Sensitivity Predicts Short-Term Outcome of Postural Tachycardia Syndrome in Children

**DOI:** 10.1371/journal.pone.0167525

**Published:** 2016-12-09

**Authors:** Hongxia Li, Ying Liao, Yuli Wang, Ping Liu, Chufan Sun, Yonghong Chen, Chaoshu Tang, Hongfang Jin, Junbao Du

**Affiliations:** 1 Department of Pediatrics, Peking University First Hospital, Beijing, P. R. China; 2 Department of Physiology and Pathophysiology, Peking University Health Sciences Centre, Beijing, P. R. China; 3 Key Laboratory of Cardiovascular Sciences, Ministry of Education, Beijing, P. R. China; University of Adelaide, AUSTRALIA

## Abstract

**Objective:**

The study was designed to examine if baroreflex sensitivity (BRS) could predict the short-term outcome of postural tachycardia syndrome (POTS) in children.

**Methods:**

Seventy-seven children subjects were included in the study. Among them, 45 children were in the POTS group and another 32 healthy children were in the control group. A ninety-day clinical follow-up was conducted and the symptom score before and after the follow-up was calculated for POTS patients by using POTS score system. Hemodynamics and continuous BRS monitoring were recorded by Finapres Medical System-FMS (FinometerPRO, FMS Company, Netherlands). According to the symptom score change during follow-up period, POTS patients were further divided into subgroup A (n = 24) with symptom score decreased by at least two points and subgroup B (n = 21) with symptom score decreased by less than two points. The predictive value of BRS in the short-term outcome of POTS in children was analyzed using receiver-operating characteristic (ROC) curve.

**Results:**

BRS of POTS children was significantly higher than that of the healthy children (18.76±9.96 ms/mmHg vs 10±5.42 ms/mmHg, P<0.01). It was higher in subgroup B than that of subgroup A (24.7±9.9 ms/mmHg vs 13.5±6.6 ms/mmHg, P <0.01). BRS was positively correlated with HR change in POTS Group (r = 0.304, P <0.05). Area under curve (AUC) was 0.855 (95% of confidence interval 0.735–0.975), and BRS of 17.01 ms/mmHg as a cut-off value yielded the predictive sensitivity of 85.7% and specificity of 87.5%.

**Conclusions:**

BRS is a useful index to predict the short-term outcome of POTS in children.

## Introduction

Postural tachycardia syndrome (POTS) is one of the subtypes of orthostatic intolerance (OI) in children. Its symptoms had significant associations with postural change. Main hemodynamic characteristics of POTS was a significant increase in heart rate at upright position compared with supine position, and the increase amplitude of heart rate is ≥40 heart beats/min, or the maximum heart rate is ≥120 heart beats/min [1]. Main clinical features are orthostatic intolerance symptoms, such as dizziness, amaurosis, chest tightness, pale complexion, blurred vision, long out-gassing and palpitation, and some children patients might even have syncope, seriously affecting daily life and academic records of children patients [2–3]. Studies showed that approx. 500,000 to 3000,000 persons suffered from POTS in the USA [4]. Low *et al*. showed that the morbidity rate of POTS was approx. 170/100,000 [5], while others showed that the morbidity rate of POTS was approx. 10% [6]. Therefore, the outcome of POTS prognosis is becoming an increasing concern nowadays. The prognosis of the disease varies depending on the different degrees of POTS condition and different predisposing factors of POTS children. However, up to now, the effective predictors of POTS prognosis in children is lacking.

Generally speaking, the POTS prognosis is related with its pathogenesis [7]. The previous research showed that the pathogenesis of POTS was mainly related to the reduction of central blood volume, autonomic reflex abnormality, endothelial dysfunction, abnormal pumping function of muscle and gene mutation, etc [8–14]. Autonomic reflex abnormality was the main pathogenesis of POTS [15–16]. When one is at upright position, returned blood volume decreases, left ventricular filling decreases, and cardiac output decreases accordingly. Via the baroreceptor at carotid sinus and aorta arch, impulse is transmitted to cardiovascular motor center, reflectively increasing the sympathetic excitability, thus resulting in an increase in ventricular contractility, increase in heart rate and peripheral vasoconstriction, maintaining normal blood pressure. In the above reflex process, baroreflex sensitivity (BRS) is critical. Zhu, *et al*. showed that POTS children had abnormality of autonomic nervous function [17]. Furlan, *et al*. showed that sympathetic activity of POTS patients increased abnormally, and the abnormality of autonomic nervous activity was involved in pathogenesis of POTS [18]. Therefore, it was speculated that the abnormality of autonomic reflex was related to disease severity of POTS patients, which would affect the disease prognosis of children patients. Thus, to further find out the markers reflecting autonomic reflex abnormality of POTS patients would be very useful to understand the prognosis of the POTS in children.

BRS comprises basic mechanistic elements of regulating blood pressure. Carotid sinus and aorta arch sense the traction of vascular wall by blood pressure change, transmitting to cardiovascular regulatory center of medulla and hypothalamus, thus to regulate the activity of autonomic nervous system. Thus, BRS plays an important role in keeping the stability of cardiovascular function, especially the regulation of upright blood pressure change. Harrington, *et al*. showed that the reduction of BRS was closely related to the occurrence of hypertension [19]. However, there was lack of studies at present on whether BRS could reflect the degree of autonomic nervous system function of POTS children and whether BRS could predict POTS outcome in children. Therefore, the present study was undertaken to examine whether BRS could be used a predictor of short-term outcome to conventional therapy in children with POTS.

## Patients and Methods

### Subjects

This research included 45 POTS children, all of whom were admitted to the Department of Pediatrics of Peking University First Hospital because of orthostatic intolerance symptom from November 2013 to August 2015. They were diagnosed as POTS according to the clinical pictures and upright tilt test [3,20]. The control group consisted of 32 healthy children on history-taking, physical examination and upright test. Cardiac, neurological and metabolic diseases that could cause orthostatic intolerance symptoms were excluded.

Medical records and samples were obtained from November 2013 to August 2015. Authors had access to information that could identify individual participants during data collection. All the selected subjects had no history of fever and medication within two weeks. This research protocol was approved by Ethics Committee of Peking University First Hospital, Beijing, China and the informed consent was obtained from parents and guardians of all study subjects. We obtained informed written consent from the next of kin, caretakers, or guardians on behalf of the children enrolled in the study. The Ethics Committee of Peking University First Hospital for Clinical Study approved this consent procedure.

### Diagnosis of POTS

(1) mainly elder children; (2) with orthostatic intolerance, such as dizziness and headache, amaurosis, palpitation, nausea, blurred vision and even syncope in severe cases, with orthostatic intolerance symptoms lasting for at least three months; (3) positive head-up tilt test (HUTT) with an increase in heart rate ≥ 40 heart beats/min or a maximum heart rate ≥120 heart beats/min when upright or tilt within 10 min, accompanied with orthostatic intolerance symptoms including dizziness and headache, amaurosis, palpitation and nausea, and blurred vision, and syncope might be cause in severe condition; (4) in addition, other diseases that could cause orthostatic intolerance symptoms including cerebral vascular diseases and organic heart disease were excluded [3,20].

#### HUTT

The protocol for HUTT was according to the previously published literatures [3, 20]. Children were required to stop all drugs that might affect autonomic nervous function before the test, and empty the bladder before the test in fasting state. The test was carried out in a quiet environment with dim light and conformable temperature. First, children were kept supine on tilt table for 10 min, and heart rate, blood pressure and ECG were monitored by using a Dash 2000 Multi-lead Physiological Monitor (General Electric, NY, New York, USA). Then, children stood on a tilt table (tilt angle of 60°), and heart rate, blood pressure and ECG were monitored, until the occurrence of positive reaction or completion of the whole test for 45 min.

### Hemodynamic data collected by Finapres Medical System-FMS

FMS (FinometerPRO, FMS Company, Netherlands) with a finger sensor was used for continuously monitoring and collecting hemodynamic data generated beat-by-beat. HR, BP and BRS were measured using a model flow method. With a finger cuff, arterial blood pressure was obtained continuously and noninvasively by using the system and beatscope software with the volume-clamp technique maintaining the diameter of the artery under an inflated finger cuff at a set point, thereby determining arterial pressure with time changes. Diodes were located in the finger cuff, on either side of the finger, to detect changes in artery diameter and change the inflation of the cuff to remain the diameter at the set point. The cuff is inflated or deflated via an air bladder connected to an air hose and pump. The software, using a mathematical model, generates an aortic pulse waveform from the finger arterial pressure wave. This computation takes into account changes in the pulse pressure and waveform shape as the pressure pulse is transmitted down the brachial arteries to the finger arteries. With the Finapres finger cuff, together with beatscope software, left ventricular stroke volume was calculated and together with HR and BRS were calculated. The BRS was calculated at rest and in supine position. Parameters such as age, sex, body height and weight were also included in the computation for each individual subject [21–23]. The measured index was derived from a mean of 3–6 cardiac cycles.

### Symptom scores

Symptom scoring was applied to evaluate the effect of conventional therapy. Score was calculated according to occurrence frequency of clinical symptom of subjects to carry out respective score of clinical symptom when diagnosed and at follow-up of patients; symptom score when diagnosed was recorded as baseline score, and symptom score at follow-up was recorded as the end score. Score calculation was also applied to control subjects. Subjects were instructed to rate their symptoms for at least 3 months prior to completion of questionnaire by parent or subjects. Score included the following clinical symptoms: syncope, dizziness, chest tightness, nausea, palpitation, headache, blurred vision, hand shaking and cold sweat. The score calculation standard was provided below: 0 score, no symptoms; 1 score, symptoms less than once on average per month; 2 scores, 2–4 times on average per month; 3 scores, 2–7 times on average per week; and 4 scores, over once on average per day. All the scores were the sum of each symptom-based score. After children were diagnosed as POTS, baseline symptom scores were recorded, and scores were recorded again at follow-up according to symptom score system. Those whose end scores at follow-up decreased by more than 2 scores compared with baseline scores were assigned into subgroup A; while those whose end scores increased or decreased less than 2 scores compared with baseline scores were assigned into subgroup B [24–27].

### Treatment and follow-up

The conventional therapy was used for each POTS child in this research after diagnosis, including oral rehydration saline (ORS), exercise of autonomic nervous function and health education. The dosage of ORS was 1 bag/day. One bag of ORS contained 0.65 g of sodium chloride, 0.725 g of sodium citrate, and 0.375 g of potassium chloride. The exercise of autonomic nervous function used in this research mainly included upright training. The method of upright training was to stand against a wall (15–20 cm from both ankles to the wall), once a day, for 5–20 min. The training time was gradually extended according to the tolerance degree of upright position. In case of syncope aura or other disorders occurred, the training was terminated immediately [28–29]. Health education was to inform parents of patients of possible causes [30–31]. The above therapies were conventional, and outpatient follow-up or telephone follow-up of 13 cases was carried out for conditions of patients subsequently, with a follow-up duration of ninety days. All subjects completed the follow-up.

### Statistical analyses

The analysis was carried out with SPSS 16.0 software, and measurement data are expressed in mean ± standard deviation. Independent sample t-test was used for comparison of BRS between two groups, and variance of analysis was used for comparison among three groups. Pearson correlation analysis was used for correlation analysis, and chi-square test was used for comparison of rates. ROC curve was used to analyze BRS on prediction of the prognosis of POTS children, and area under curve indicated the value of the index on prediction of the result; area under curve between 0.5–0.7 indicated a relatively small predictive value, area under curve between 0.7–0.9 indicated a medium predictive value, and area under curve above 0.9 indicated a relatively high diagnostic value. Ninety-five percent of confidence interval of the curve area (not including 0.5 or p<0.05) indicated that the index had a value on prediction of the research result. P<0.05 indicated there was statistical difference.

## Results

### Baseline characteristics of the POTS group and control group

The POTS group included 25 males and 20 females, at the age of 12.5±2.5 years old. Control group consisted of 19 males and 13 females, at the age of 11.8±3 years old. Two groups had no statistical difference (p>0.05) in gender ratio, age, height, weight, systolic pressure at supine position, and diastolic pressure at supine position. The heart rate of POTS children at supine position, however, was lower than that of children in control group (p<0.05), and BRS value of POTS group was higher than that of children in control group (p<0.01) (Table 1).

**Table 1:** Baseline characteristics of the POTS group and control group.

Characteristics	Control Group	POTS Group	t /χ^2^	p value
Cases, n	32	45	-	-
male/female	19/13	25/20	0.338	0.561
Age, yrs	11.8±3	12.5±2.5	-1.056	0.294
Height, cm	155.7±14.5	156.8±13.4	-0.361	0.719
Weight, kg	50.5±12.6	47.3±12.5	1.099	0.275
Supine systolic BP, mmHg	109.6±13.9	115±16.6	-1.519	0.133
Supine diastolic BP, mmHg	67.9±9	71.3±10.4	-1.495	0.139
Supine heart rate, beats/min	85.3±11.1	77.7±14.5	2.473	0.016
Supine BRS, ms/mmHg	10.0±5.4	18.8±9.9	-4.523	0.001

BP: blood pressure; BRS: baroreflex sensitivity

### Comparison of symptom score

The end symptom score at follow-up was lower than the baseline symptom score when diagnosed (p<0.01) in POTS group (Table 2). The baseline symptom score of the controls was zero.

**Table 2:** Comparison of symptom scores in POTS children.

Time	n	Symptom score
First visit	45	4.67±1.28
The end of follow-up	45	2.73±1.12
t	-	9.691
p value	-	0.0001

### Baseline characteristics of the subgroup A and subgroup B

No statistical difference were found in gender ratio, age, height, weight, heart rate, systolic pressure at supine position, and diastolic pressure at supine position between the subgroup A and subgroup B (p>0.05) (Table 3). There was no statistical difference in baseline symptom score between the subgroup A and subgroup B (p>0.05), but the end symptom score of subgroup A was lower than that of subgroup B at follow-up (p<0.01) (Table 4).

**Table 3:** Baseline characteristics of the group A and group B.

Characteristics	Subgroup A	Subgroup B	t /χ^2^	p value
Cases, n	24	21	-	-
male/female	12/12	11/10	0.025	0.873
Age, yrs	13±2.4	11.8±2.4	-1.775	0.083
Height, cm	160±8.8	154±16.9	-1.416	0.164
Weight, kg	47.9±10.4	46.7±14.8	-0.321	0.750
Supine systolic BP, mmHg	119±15	110.9±17	-1.994	0.053
Supine diastolic BP, mmHg	73.3±9.7	69±11	-1.389	0.172
Supine heart rate, beats/min	81.2±13	73.8±15.4	-1.752	0.087
Supine BRS, ms/mmHg	13.5±6.6	24.7±9.9	4.517	0.001

BP: blood pressure; BRS: baroreflex sensitivity

**Table 4:** Symptom scores between subgroup A and subgroup B in POTS.

Groups	n	Symptom score at first visit	Symptom score at the end of follow-up
Subgroup A	24	4.88±1.23	2.21±0.8
Subgroup B	21	4.43±1.33	3.67±1.23
t	-	-1.173	4.687
P value	-	0.247	0.0001

POTS: postural tachycardia syndrome

### Comparison of BRS between subgroup A and subgroup B

**Fig 1:**
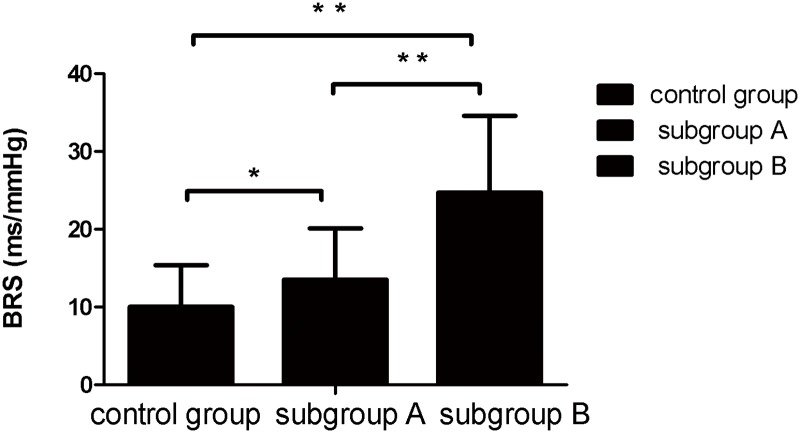
BRS among children in subgroup A and B, and controls. Compared with that of control group, BRS was increased in subgroups A and B (P <0.05, P <0.01, respectively). BRS of subgroup B was higher than that of subgroup A (24.7±9.9 ms/mmHg vs 13.5±6.6 ms/mmHg, P <0.01).

BRS of POTS children was significantly higher than that of the healthy children (18.76±9.96 ms/mmHg vs 10±5.42 ms/mmHg, P<0.01). It was higher in subgroup B than that of subgroup A (24.7±9.9 ms/mmHg vs 13.5±6.6 ms/mmHg, P <0.01) (Fig 1).

**Fig 2:**
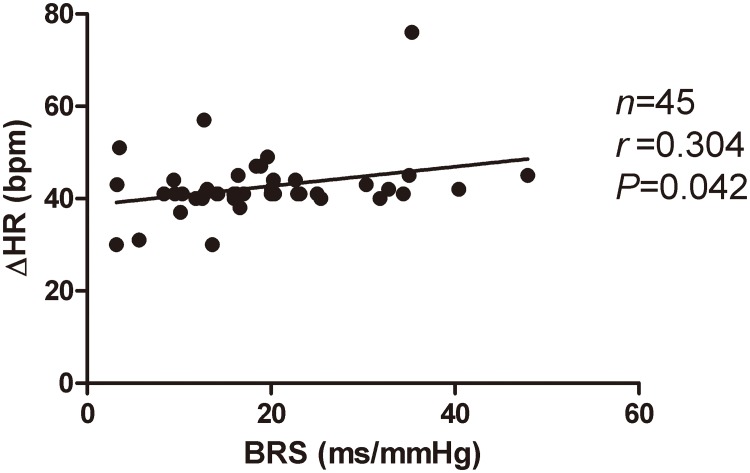
relation of heart rate change from supine to upright with baseline BRS in POTS children. Cor Heart rate change from supine to upright was positively correlated with BRS in POTS group (r = 0.304, P <0.05).

Correlation analysis of BRS and HR in POTS group: Heart rate change from supine to upright was positively correlated with BRS in POTS group (r = 0.304, p<0.05, Fig 2).

Score in group A had no correlation with BRS (P>0.05).

**Fig 3:**
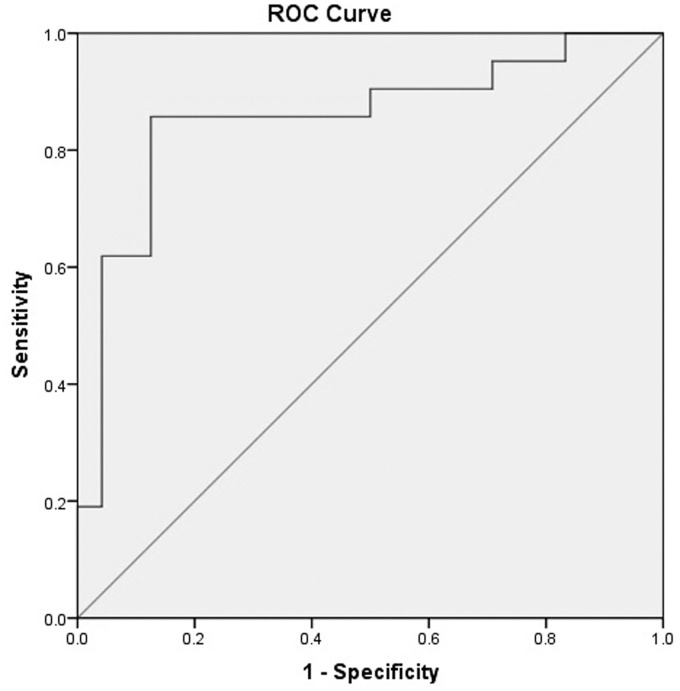
ROC analysis of baseline BRS prediction of short-term outcome of POTS patients. With BRS value of 17.01 ms/mmHg as a cutoff value, the predictive sensitivity was 85.7%, and specificity 87.5%.

ROC curve of the predictive value of BRS for short-term outcome of POTS: The ROC curve showed that the area under the curve was 0.855 (95%CI 0.735–0.975). With BRS value of 17.01 ms/mmHg as a cutoff value, the predicted sensitivity was 85.7%, and specificity 87.5% (Fig 3).

## Discussion

POTS has a relatively high incidence among school-aged children, with the proportion of girls higher than boys [32]. In China, studies showed that POTS children accounted for 28.8% of childhood syncope [33]. Half of POTS patients could not regularly attend school due to chronic orthostatic intolerance, and 30%-40% of children with POTS were unable to regularly attend school [34]. POTS seriously affects daily life, learning quality and social adaptability of children, causing the reduction of academic achievement [35–39]. Low, *et al*. showed that POTS patients were characterized by intolerance to movement and physical labor in addition to orthostatic intolerance [6]. Therefore, the disease prognosis of POTS became a hot issue in pediatrics. However, there was lack of knowledge of the prognosis prediction of POTS patients.

The pathogenesis of POTS mainly involved autonomic nervous dysfunction [15]. BRS plays an important role in instantaneous regulation of blood pressure. Under normal condition, when the human body changes from supine to upright position, approx. 500–1000 ml of blood are transferred to abdomen, pelvis and lower extremities because the blood moves down due to gravity, resulting in a reduction of returned blood volume, and a decrease in cardiac output. The impulse introduced into carotid sinus and aorta arch baroreceptor is reduced, causing an increase in reflectivity of heart rate and increase in cardiac output through regulation of vascular center, thus to make blood pressure recover to normal level. This is a normal pressor reflex. However, the increase range of heart rate of healthy people was 10–15 heart beats/min, which reaches the upright stable state again within 1 min [40–41]. When children with POTS change from supine to upright position, however, the increase value of heart rate was ≥40 heart beats/min. However, whether autonomic reflex abnormality exists in pressor process of POTS children to cause an over increase in heart rate at upright position has not been clear.

The present study showed that BRS of children with POTS was significantly higher than that of control subjects (18.76±9.96 ms/mmHg vs 10±5.42 ms/mmHg, P <0.01). Convertino, *et al*. showed that low blood volume might cause increase in BRS [42]. When POTS patients change to upright position from supine position, returned blood volume decreases, and cardiac output decreases; at this time, if BRS increases abnormally, it would cause an over excitation caused by sympathetic nervous system reflectively when blood pressure of patients decreases slightly, thus to cause an over increase in heart rate of children at upright position. In addition, the present study showed that BRS was positively correlated to an increase in heart rate of children suffering from POTS from supine to upright position (r = 0.304, P <0.05), suggesting that abnormal increase in BRS might participate in the pathogenesis of POTS. However, BRS varied with age [43]. In adult POTS patients, BRS did not change as compared with normal controls demonstrated by Galbreath, *et al* [44] and Fu, *et al* [45]. While, in adolescent POTS cases (mostly white patients) aged 12–17 (median 15.2) years old, baroreceptor attenuation was observed through defective vagal efferent response indirectly obtained by calculating R-R interval and heart rate variability using an arterial tonometer and continuous electrocardiographic recorder [46]. But in younger children at the mean age of 12.5 years old, the present study showed that BRS was increased as compared with controls. Therefore, further exploring the age-based developmental regularity of baroreceptor function would be of great value in a better understanding of pathogenesis of POTS.

The present follow-up study showed that BRS of POTS in subgroup A was significantly lower than that in subgroup B (14.3±6 ms/mmHg vs 21.4±11.2 ms/mmHg, P <0.01). It was speculated that abnormal increase in BRS might affect disease prognosis. POTS patients with high BRS had a low threshold of blood pressure decrease caused by position change, and slight change in pressure might reflectively cause over activation of sympathetic nerve of children cases, resulting in a greater adverse impact on the prognosis if the increase value of heart rate was increased. As such, the present study suggested that baseline BRS might be a good index of predicating the prognosis of children with POTS, and the area under curve was 0.855, with a BRS value of 17.01 ms/mmHg as a cutoff value yielding the predicted sensitivity of 85.7% and specificity of 87.5%. Hence, detection of BRS could well predict the disease outcome of POTS patients, and the advantage of the detection was convenient, inexpensive, easy to perform and noninvasive in the prediction.

The accuracy of the finger monitoring method of hemodynamics used in present study has been proved in previous studies and it can well reflect the real hemodynamics [47–50]. However, this study still has limitations. For ethic and safety reasons, HUTT was not performed in children of control group. Therefore, the BRS data in upright position were not collected for controls. It was not clear whether BRS could predict outcome based on tilt response, which was the limitation of this study. The sample size in this research is relatively small, which might probably be one of the reasons for the fact that the majority of patients were male. But no study showed any relationship between BRS with gender. In some cases, scoring was done over the phone, which might induce a bias in the analysis. Further follow-up studies are necessary to improve the value of BRS in the prediction of long-term prognosis of POTS patients.

In conclusion, BRS value is a useful index to predict the short-term outcome of POTS in children.
